# Mansouramycin C kills cancer cells through reactive oxygen species production mediated by opening of mitochondrial permeability transition pore

**DOI:** 10.18632/oncotarget.22004

**Published:** 2017-10-24

**Authors:** Shan Kuang, Ge Liu, Ruobing Cao, Linlin Zhang, Qiang Yu, Chaomin Sun

**Affiliations:** ^1^ Key Laboratory of Experimental Marine Biology, Institute of Oceanology, Chinese Academy of Sciences, Qingdao, China; ^2^ Laboratory for Marine Biology and Biotechnology, Qingdao National Laboratory for Marine Science and Technology, Qingdao, China; ^3^ College of Earth Science, University of Chinese Academy of Sciences, Beijing, China; ^4^ Division of Tumor Pharmacology, Shanghai Institute of Materia Medica, Chinese Academy of Sciences, Shanghai, China

**Keywords:** mansouramycin C, marine-derived isoquinolinequinone, reactive oxygen species, mitochondrial permeability transition pore, anticancer drug

## Abstract

Cancer is one of the deadliest diseases in the world and the search for novel anticancer agents is urgently required. Marine-derived isoquinolinequinones have exhibited promising anticancer activities. However, the exact mechanisms of cytotoxic activities of these isoquinolinequinones are poorly characterized. In this study, we investigated the anticancer effects and molecular mechanisms of mansouramycin C (Mm C), a cytotoxic isoquinolinequinone isolated from a marine streptomycete. We demonstrated that Mm C preferentially killed cancer cells and the cytotoxic effects were mediated by reactive oxygen species (ROS) generation. Mass spectrometry based proteomic analysis of Mm C-treated A549 cells revealed that many ROS-related proteins were differentially expressed. Proteomic-profiling after Mm C treatment identified oxidative phosphorylation as the most significant changes in pathways. Analysis also revealed extensive defects in mitochondrial structure and function. Furthermore, we disclosed that Mm C-induced ROS generation was caused by opening of mitochondrial permeability transition pore. Notably, Mm C synergized with sorafenib to induce cell death in A549 cells. Hence, we propose that the marine-derived natural compound Mm C is a potent inducer of the mitochondrial permeability transition and a promising anticancer drug candidate. Moreover, molecular mechanisms of Mm C shed new light on the understanding of the cytotoxic mechanisms of marine-derived isoquinolinequiones.

## INTRODUCTION

Cancer is a major cause of morbidity and mortality with approximately 14 million new cases and 8 million cancer-related deaths in 2012 worldwide [1]. It is estimated that compared with 2012, the annual number of new cancers will grow by 70% by 2035 [1]. Lung cancer remains the most common cancer in 2012 worldwide, both in term of new cases (1.8 million cases, 12.9% of total) and deaths (1.6 million deaths, 19.4% of total) [2]. Thus, the search for more effective anticancer drugs, especially lung cancer drugs, is urgently required.

Natural products have long been an important source of anticancer drugs. A detailed analysis of anticancer drugs approved from around the 1940s to 2014 revealed that of the 175 small molecules approved 85, or 49%, were either natural products or their derivatives [3]. Marine biodiversity is extremely rich. The described marine species are approximately 230,000 to 275,000 and the inventory is accruing 1,300 to 1,500 species per year [4], which indicates a striking potential for anticancer drug discovery. However, marine biodiversity is poorly explored and discovery of marine natural compounds has only begun: approximately 22,000 natural products of marine origin have been discovered so far, whereas 131,000 terrestrial natural products exist [5]. Also, marine natural compounds which have either been marketed or under development are relatively few. To date, there are only seven approved marine-derived pharmaceuticals in clinical use and four of them are anticancer drugs [6]. It might therefore be attractive to further explore marine natural compounds for anticancer drug development.

Selectivity remains a big challenge in cancer treatment. Accumulating evidence suggests that increase of reactive oxygen species (ROS) is effective for selective cancer therapy [7–9]. Compared with their normal counterparts, most cancer cells are under oxidative stress with increased production of ROS [10, 11]. Severe increase of ROS could cause irreversible oxidative damage of cells leading to cell death [12]. When both cancer and normal cells are exposed to equal intensity of exogenous ROS stimulating agents, the ROS levels in cancer cells would be more easier to reach a threshold to trigger death [13]. The vast majority of anticancer drugs such as ionizing radiation agents, most chemotherapeutic agents and some targeted agents kill their target cells directly or indirectly through the generation of elevated amounts of intracellular ROS [14]. Many ROS inducers are currently under various stages of drug development [12]. Among them, clinical use of As_2_O_3_ in acute promyelocytic leukemia (APL) has advanced to first-line therapy [15].

Mitochondrial energy metabolism is the most quantitatively important source of intracellular ROS under physiological conditions [16]. In addition to their established role in energy metabolism, mitochondria are main actors in cell death [17]. In this context, permeabilization of the inner mitochondrial membrane- an event regulated by the mitochondrial permeability transition pore (PTP) - plays a central role in cell death [18, 19]. PTP is a multiprotein complex built up among membranes of mitochondria and allows molecules with a molecular weight of less than 1.5 kD to pass freely into and out of the inner membranes of mitochondria [19, 20]. Opening of PTP causes mitochondrial permeability transition (MPT), which leads to dissipation of mitochondrial membrane potential (MMP), uncoupling of oxidative phosphorylation and increase in mitochondrial volume (swelling). The PTP open-closed transition is modulated by a variety of factors. Ca^2+^ and oxidant stress are key factors of MPT induction [21, 22], while the PTP inhibitor cyclosporin A (Cs A) has become the standard diagnostic tool for the characterization of the PTP [23].

Mansouramycin C (Mm C, 3-carbomethoxy-7-methylaminoisoquinoline-5, 8-dione) is a natural isoquinolinequinone isolated from marine-derived *Streptomyces* sp. isolate Mei37 [24]. Among the four isolated mansouramycins (mansouramycin A-D), Mm C is the most active cytotoxic compound, with a mean EC_50_ value of 89 nM against 36 tumor cell lines tested [24]. However, the molecular targets and mode of action of Mm C remain unclear. Many marine-derived isoquinolinequinones, including renierone, cribrostatins, perfragilins and caulibugulones, are attractive due to their anticancer properties [25–28]. Nevertheless, the exact mechanisms of action of these marine-derived cytotoxic isoquinolinequinones are poorly characterized [29, 30]. Thus, elucidation of the molecular mechanisms of Mm C will be helpful to understand the cytotoxic mechanisms of these isoquinolinequiones.

In the present study, we synthesized Mm C and investigated the molecular targets and mode of action of it. It preferentially killed cancer cells through induction of ROS. In addition, Mm C caused functional and structural defects of mitochondria. Finally we demonstrated that Mm C induced ROS production through opening of mitochondrial PTP. Notably, Mm C synergized with sorafenib to inhibit cancer cell growth. Our data strongly supports the notion that Mm C is a novel inducer of MPT and is a promising anticancer drug candidate.

## RESULTS

### Mm C preferentially kills cancer cells

**Figure 1:**
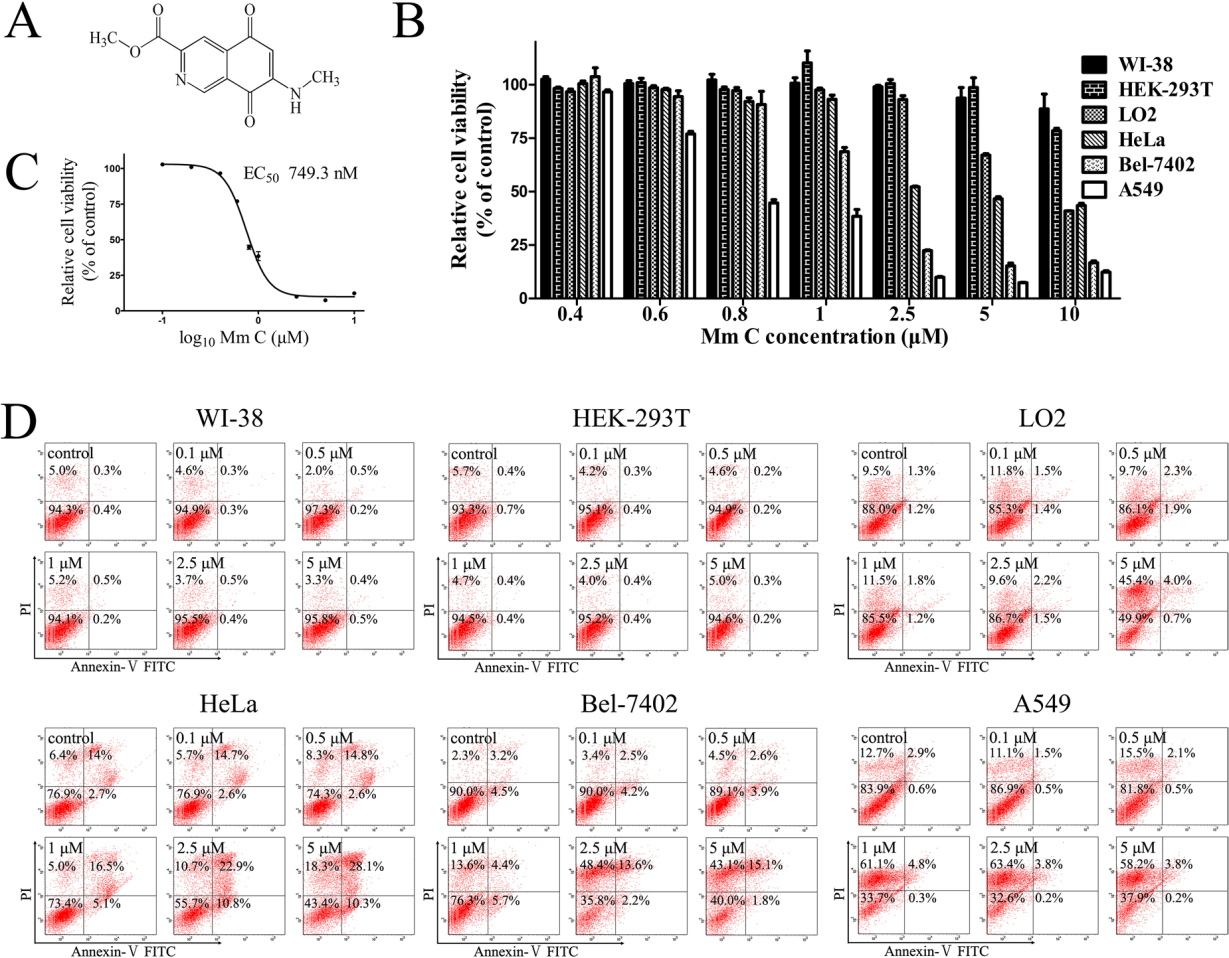
Mm C preferentially killed cancer cells **(A)** Chemical structure of Mm C. **(B)** Distinct cytotoxic effects of Mm C on normal cell lines Wi-38, HEK-293T and LO2 and cancer cell lines HeLa, Bel-7402 and A549 for 6 h determined by MTT assay. **(C)** EC_50_ of Mm C on viability of A549 cells for 6 h determined by MTT assay. **(D)** Flow cytometric analysis of Mm C-treated cells. Different normal cells as well as cancer cells were pretreated with indicated concentrations of Mm C for 6 h. Cells were then stained with Annexin V-FITC and PI before analysis of cell death through flow cytometry.

Mm C (Figure 1A) is a natural isoquinolinequinone isolated from a marine streptomycete with potent cytotoxic activity [24]. To investigate its mode of action and therapeutic potential, we tested the effects of Mm C on human cancer and normal cells. Interestingly, Mm C preferentially killed cancer cells including human lung cancer cells A549, liver cancer cells Bel-7402 and cervical cancer cells HeLa compared with normal cells including human embryonic lung fibroblasts WI-38, liver cells LO2 and embryonic kidney cells HEK-293T. As shown in Figure 1B, treatment with 2.5 μM Mm C for 6 h caused about 50% or more decrease of the MTT value of cancer cell lines, whereas it had very little growth inhibition effect on normal cell lines.

We chose the most sensitive cell line A549 as our model to investigate the molecular mechanisms of Mm C. In the MTT assay, the growth inhibitory effect of Mm C on A549 cells for 6 h was concentration-dependent, with a 50% inhibitory concentration value of 749.3 nM (Figure 1C). In the trypan blue exclusion staining assay, Mm C also dose-dependently inhibited the growth of A549 cells with an EC_50_ of 814.8 nM (Supplementary Figure 1A). Also, we treated cells with Mm C for 6 h, then removed Mm C and incubated cells with fresh medium for another 24 h. Cell viability was determined by MTT assay and the EC_50_ was calculated to be 457.0 nM (Supplementary Figure 1B), which is lower than the EC_50_ of Mm C for 6 h, suggesting that Mm C might cause cell death of A549 cells.

Annexin V-FITC/ propidium iodide (PI) double-staining assays showed that treatment of Mm C for 6 h dose-dependently caused cell death of cancer cells (Figure 1D). As shown in Figure 1D, for HeLa cells, Mm C mainly caused apoptosis; for Bel-7402 cells, Mm C mainly caused necrosis while for A549 cells, Mm C only caused necrosis. As for normal cells, 5 μM Mm C caused necrosis of LO2 cells while it had little effects on WI-38 and HEK-293T cells (Figure 1D). Taken together, these data showed that Mm C selectively killed cancer cells.

### ROS contributes to the cytotoxic effects of Mm C

**Figure 2:**
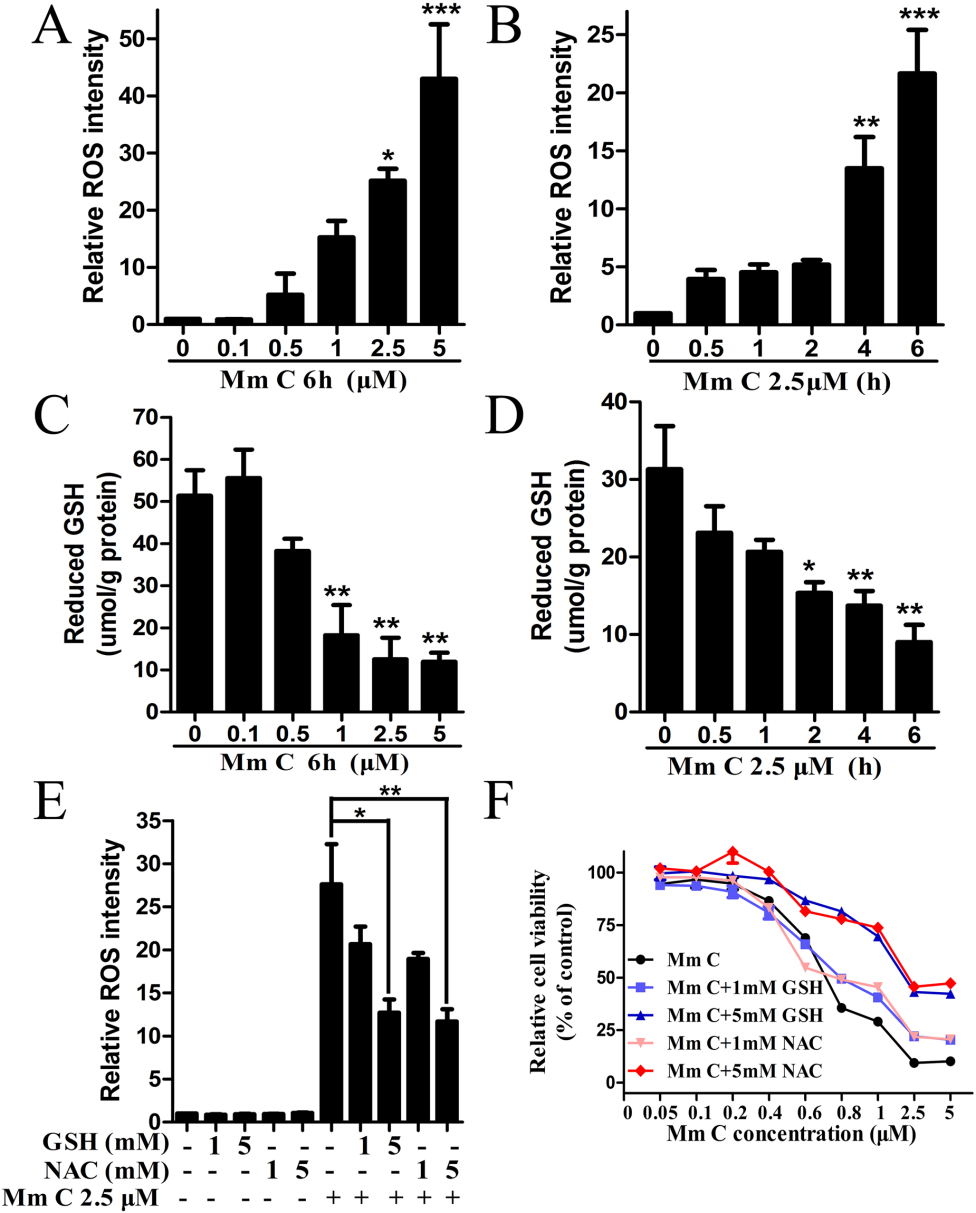
ROS contributed to the cytotoxic effect of Mm C **(A, B)** Mm C dose- (A) and time-dependently (B) induced accumulation of ROS. A549 cells were treated with Mm C and then ROS levels were measured by flow cytometry. ^*^P < 0.05, ^**^P < 0.01, ^***^P < 0.001 compared with control. **(C, D)** Mm C dose- (C) and time-dependently (D) decreased intracellular GSH levels. A549 cells were treated with Mm C and then intracellular GSH levels were measured by a DTNB reaction kit and quantified by protein levels measured using the Bradford method. ^*^P < 0.05, ^**^P < 0.01 compared with control. **(E)** ROS scavengers reduced Mm C-induced ROS formation. A549 cells were pretreated with the indicated concentrations of GSH or NAC for 1 h and then Mm C was added for another 4 h. The ROS levels were measured by flow cytometry. ^*^P < 0.05, ^**^P < 0.01. **(F)** ROS scavengers prevented the cytotoxic effect of Mm C. A549 cells were pretreated with the indicated concentrations of GSH or NAC for 1 h and then the indicated concentrations of Mm C were added for another 6 h. Cell viability was measured using the MTT method.

ROS have been proven to be effective for selective cancer therapy according to the threshold theory [13]. Therefore, we examined the effects of Mm C on intracellular ROS levels of A549 cells using flow cytometry and a fluorescent probe, 2’, 7’-dichlorofluorescin diacetate (DCFH_2_-DA). Indeed, Mm C induced accumulation of intracellular ROS in a dose- (Figure 2A) and time-dependent (Figure 2B) manner. Notably, Mm C had extremely strong ROS stimulation potency. As shown in Figure 2A, treatment with 5 μM Mm C for 6 h resulted in a 43-fold increase of ROS-associated fluorescence intensity in A549 cells. Also, intracellular ROS levels continued to increase until 6 h after Mm C treatment (Figure 2B). While compared with A549 cells, Mm C induced much less ROS generation in normal cells (Supplementary Figure 2).

Intracellular glutathione (GSH) is the major antioxidant reagent in cells which plays major roles to maintain redox status and defend against oxidative stress. Therefore, we investigated the roles of GSH in Mm C-mediated ROS formation. As shown in Figure 2C and 2D, Mm C dose- and time-dependently decreased the intracellular levels of GSH in A549 cells. The time course of ROS production synchronized with that of GSH reduction, suggesting that these two events occurred simultaneously (Figure 2B and 2D).

To further establish the correlation between Mm C-induced ROS generation and cell growth inhibition, we investigated the effects of ROS scavengers on Mm C-treated A549 cells. Pretreatment of A549 cells with GSH and N-acetyl-L-cysteine (NAC) partially abolished the Mm C-induced elevation of ROS in a dose-dependent manner (Figure 2E). Also, both GSH and NAC dose-dependently abrogated Mm C-mediated growth inhibition of A549 cells (Figure 2F). However, 5 mM GSH or NAC cannot totally abolish the elevation of ROS or inhibition of cell growth caused by Mm C (Figure 2E and 2F), probably due to the extremely strong ROS stimulation potency of Mm C. These data indicated that accumulation of intracellular ROS contributed to the cytotoxic effects of Mm C.

### Proteomic analysis of differentially expressed proteins after Mm C treatment

**Figure 3:**
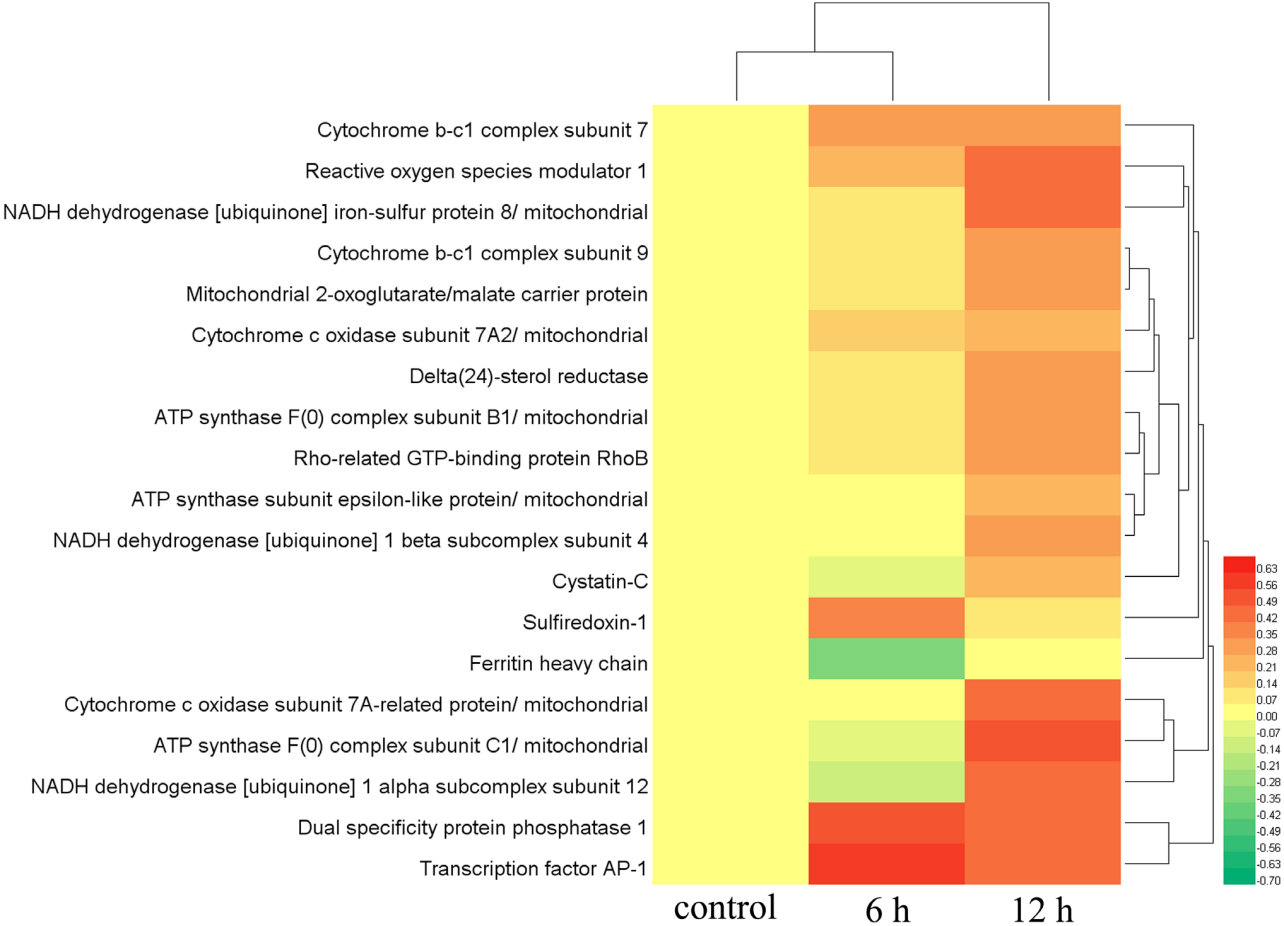
Proteomic, clustering and heatmap analysis of differentially expressed ROS-related proteins after Mm C treatment A549 cells were treated with 1 μM Mm C for 6 h or 12 h and then proteins extracted from whole cell lysates were separated and identified using LC–ESI–MS/MS analysis. The relative protein abundances of differentially expressed ROS-related proteins (fold change ≥1.2 or fold change ≤0.83 and p value <0.05) were imported for clustering analysis using HemI. Hierarchical clustering of proteins was displayed by average linkage and euclidean distance metrics and color scales of log2 values were shown. Each line represented one protein.

To better describe the contributions of ROS induction to the effects of Mm C, we performed a proteomic study to identify differentially expressed proteins after Mm C treatment. We treated A549 cells with 1 μM Mm C for 6 h and 12 h respectively and found that 89 and 134 proteins were differently expressed (1.2-fold change cutoff and p value less than 0.05, Supplementary Table 1 and 2). All these differently expressed proteins were screened to identify ROS-related proteins and 19 proteins were filtered and clustered (Figure 3). In detail, 10 of the 19 proteins are subunits of complex I, III, IV of mitochondrial electron transport chain (ETC) as well as complex V and were up-regulated after Mm C treatment. These results agree well with previous study that oxidative stress plays a critical role in the increase of mitochondria abundance of human cancer cells [31]. Reactive oxygen species modulator 1, which is known to induce ROS production [32], were up-regulated. While ferritin heavy chain was down-regulated, well-correlated with previous study that down-regulation of ferritin heavy chain increases oxidative stress [33]. Three ROS scavengers, delta(24)-sterol reductase, 2-oxoglutarate/malate carrier protein and sulfiredoxin-1, were up-regulated [34–36]. Cystatin-C, rho-related GTP-binding protein Rho B, dual specificity protein phosphatase 1 and transcription factor AP-1, which act downstream of ROS production [37–40], were up-regulated.

**Figure 4:**
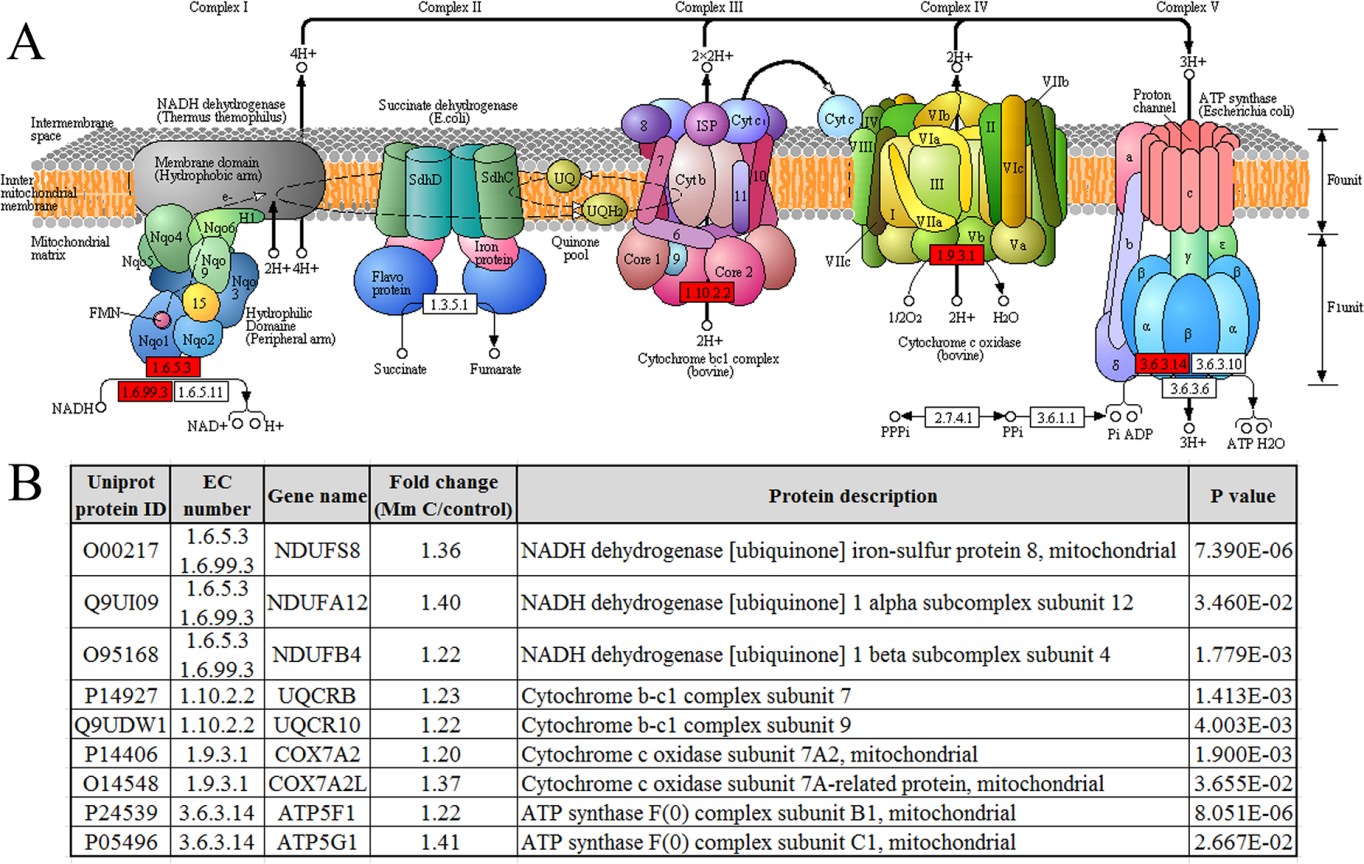
Proteomic and pathway analysis of differentially expressed proteins after Mm C treatment using the Kyoto Encyclopedia of Genes and Genomes (KEGG) Pathway database **(A)** A549 cells were treated with 1 μM Mm C for 12 h and then altered proteins were depicted in oxidative phosphorylation pathway. Up-regulated (fold change ≥1.2 and p value <0.05) or non-regulated proteins were shown in red or white, respectively. **(B)** List of the altered proteins after Mm C treatment which related to oxidative phosphorylation.

To illustrate pathways involved in the effects of Mm C. Kyoto Encyclopedia of Genes and Genomes (KEGG) orthology terms were assigned to the proteomic datasets by the KAAS sever and the KEGG mapper to map the pathways (Figure 4A). The most significant changes in pathways after Mm C treatment involved oxidative phosphorylation (p value 0.0017), which was induced in the presence of Mm C (Figure 4A and 4B). The pathway analysis confirmed the heatmap analysis of the differently expressed ROS-related proteins after Mm C treatment, suggesting that ROS is the major effect of Mm C-treated A549 cells.

### Mm C caused functional and structural defects of mitochondria

**Figure 5:**
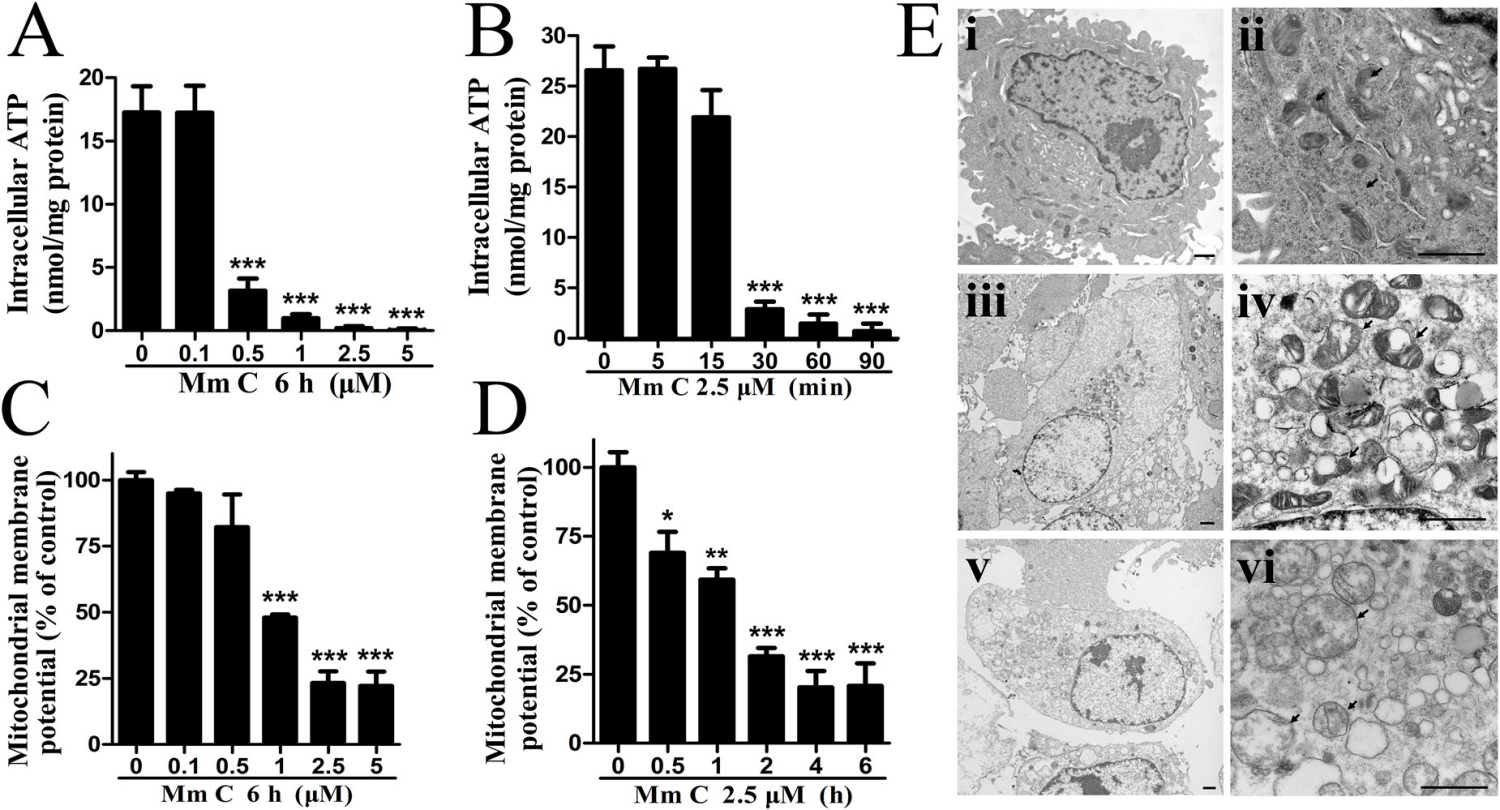
Mm C caused functional and structural defects of mitochondria **(A, B)** Mm C dose- (A) and time-dependently (B) decreased intracellular ATP levels. A549 cells were treated with Mm C and then intracellular ATP levels were determined by a chemiluminescence assay and quantified by protein levels measured using the Bradford method. ^***^P < 0.001 compared with control. **(C, D)** Mm C dose- (C) and time-dependently (D) decreased mitochondrial membrane potential (MMP). A549 cells were treated with Mm C and then the MMP was measured by fluorescent probe JC-1 and flow cytometry. ^*^P < 0.05, ^**^P < 0.01, ^***^P < 0.001 compared with control. **(E)** Mm C caused mitochondrial structural defects. A549 cells (i, ii) were treated with 2.5 μM Mm C for 2 h (iii, iv) or 6 h (v, vi) and then cells were harvested, fixed, and photographed by transmission electron microscopy. ii, iv and vi are high-power images for i, iii and v, respectively. Arrows indicate mitochondria and black scale bar is 1 μM.

Proteomic analysis of differentially expressed proteins after Mm C treatment indicated that mitochondrial complex proteins, which are the main actors in ATP production, were significantly elevated. We therefore examined the effects of Mm C on intracellular ATP levels. As shown in Figure 5A and 5B, Mm C dose- and time-dependently decreased the intracellular ATP levels in A549 cells. Since mitochondrial membrane potential (MMP) is known to be essential for the production of ATP via oxidative phosphorylation. We analyzed the effects of Mm C on MMP of A549 cells and found it dose- and time-dependently declined (Figure 5C and 5D). The decrease of intracellular ATP levels and MMP in A549 cells after Mm C treatment indicate defects in mitochondrial function.

Next, the ultrastructural morphology of mitochondria in Mm C-treated A549 cells was observed by transmission electron microscopy. As shown in Figure 5E, control cells (i, ii) contained small, morphologically normal, round or oval mitochondria with regular distribution of cristae. While Mm C-treated cells (iii, iv and v, vi) showed enlarged mitochondria with cristal disorganization (iv) or loss (vi), outer membrane buckling and distorted shape. These data indicated that Mm C caused structural defects of mitochondria in A549 cells.

**Figure 6:**
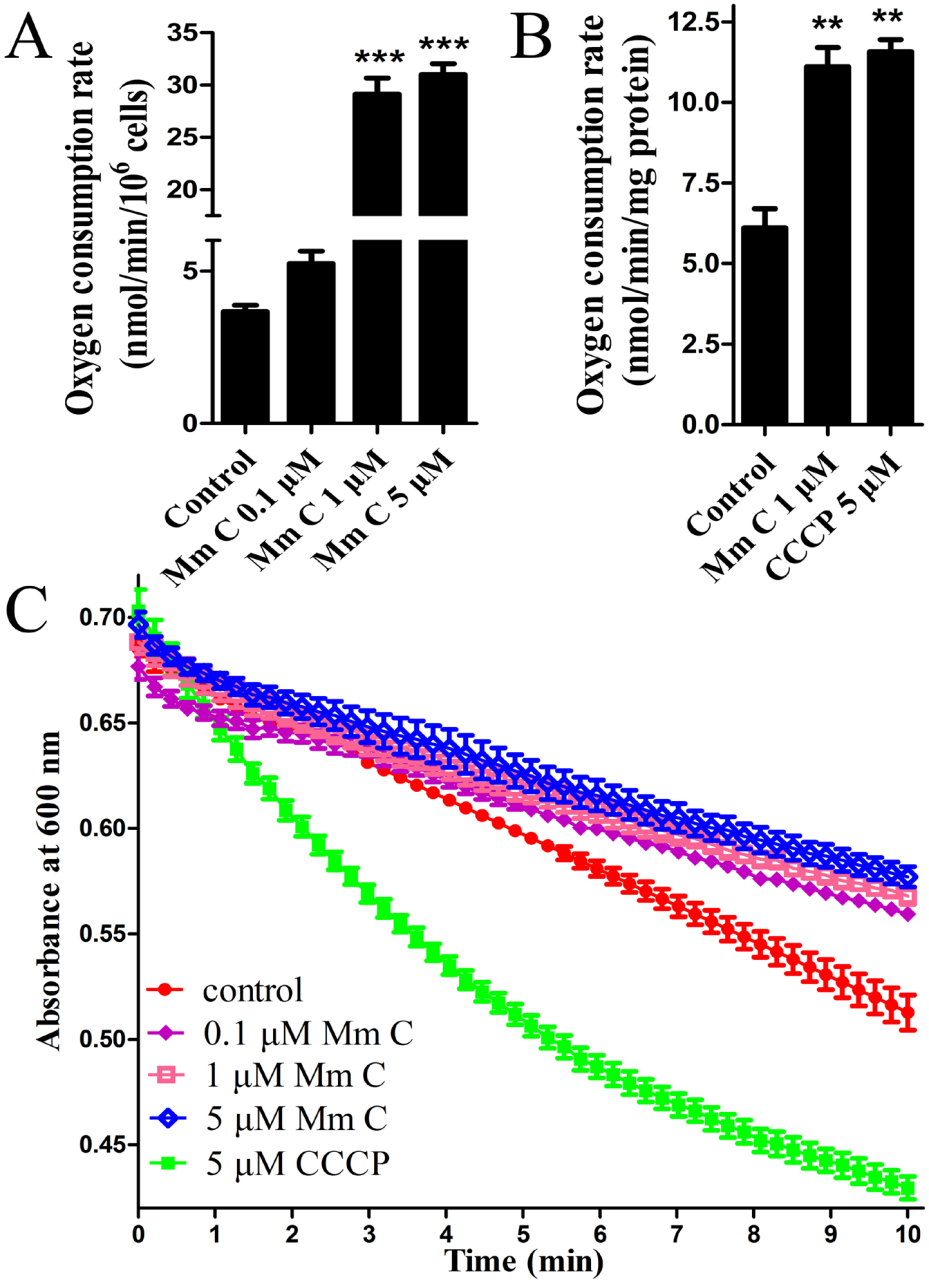
Mm C stimulated oxygen consumption rate (OCR) of cells and mouse liver mitochondria **(A)** Mm C stimulated OCR of A549 cells. OCR of A549 cells treated with indicated concentrations of Mm C was measured using a Clark-type oxygen electrode (Hansatech, King's Lynn, Norfolk, UK). ^***^P < 0.001 compared with control. **(B)** Mm C stimulated OCR of isolated mitochondria energized with glutamate and malate. OCR of mouse liver mitochondria treated with Mm C or CCCP was measured. ^**^P < 0.01 compared with control. **(C)** Unlike CCCP, Mm C did not induce proton-dependent mitochondrial swelling. Isolated mouse liver mitochondria were treated with Mm C or CCCP in hyposmotic potassium acetate medium and then absorbance at 600 nm was recorded over 10 min.

The effects of Mm C on mitochondrial function were also assessed by monitoring the respiration rate of cells as well as isolated mouse mitochondria. Oxygen consumption rate (OCR) was detected using a Clark electrode. As shown in Figure 6A, Mm C dose-dependently increased respiration rate of A549 cells. Also, we determined whether Mm C acted directly on mitochondria, we isolated mouse liver mitochondria and found that 1 μM Mm C significantly stimulated respiration rate of mitochondria (Figure 6B). Mitochondrial uncoupler carbonyl cyanide m-chlorophenyl hydrazone (CCCP), which results in respiration proceeding without phosphorylation, also stimulates mitochondrial OCR. So we compared Mm C with CCCP and found that 1 μM Mm C increased mitochondrial OCR to a similar degree as 5 μM CCCP (Figure 6B).

To determine whether Mm C transported protons across the mitochondrial inner membrane as CCCP did, we performed mitochondrial swelling assays in isotonic potassium acetate buffer supplied with the potassium ionophore valinomycin. We first confirmed the necessary role of valinomycin in CCCP induced proton-dependent mitochondrial swelling (Supplementary Figure 3). As shown in Figure 6C, CCCP induced mitochondrial swelling represented by the downward deflection of absorbance at 600 nm. However, Mm C had no effects on mitochondrial swelling (Figure 6C), indicating that Mm C stimulated mitochondrial respiration not through a protonophoric mechanism.

### Mm C induced ROS production in mouse liver mitochondria through opening of mitochondrial permeability transition pore (PTP)

**Figure 7:**
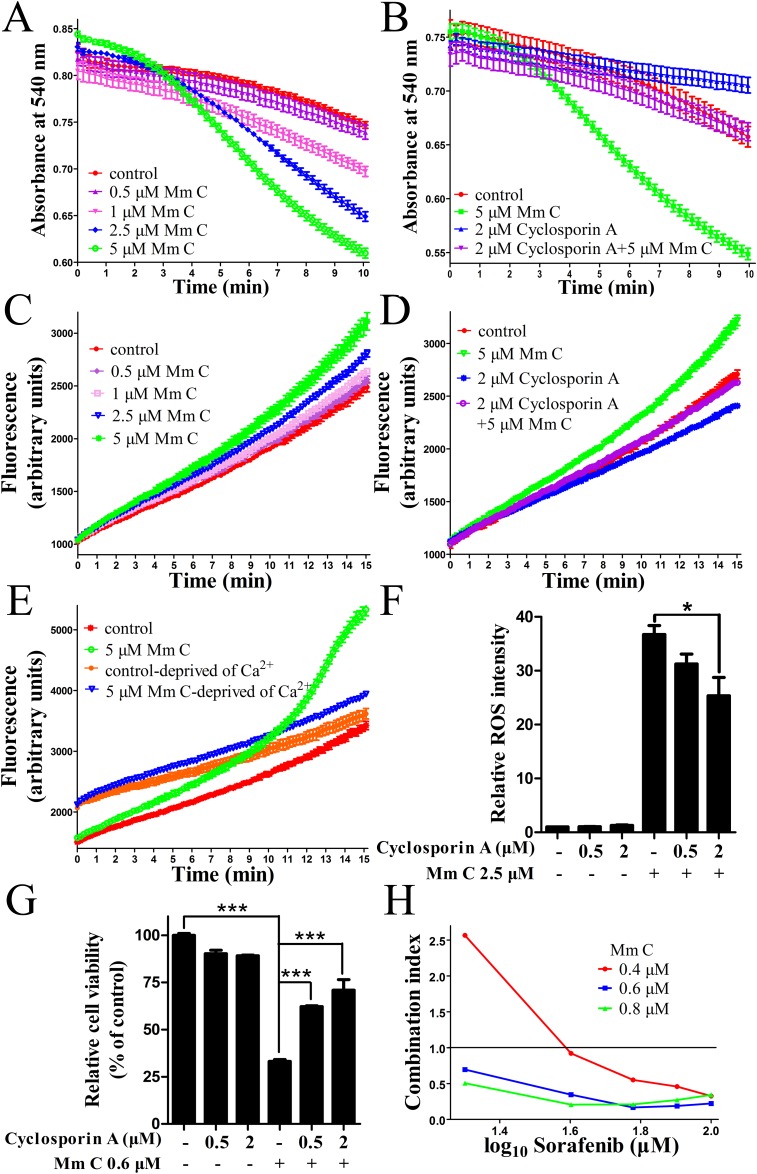
Mm C induced ROS production through opening of mitochondrial permeability transition pore (PTP) **(A)** Mm C dose-dependently induced mitochondrial permeability transition (MPT) in isolated mitochondria. Mouse liver mitochondria incubated with Ca^2+^ were treated with indicated concentrations of Mm C and then absorbance at 540 nm was recorded over 10 min. **(B)** The induction of MPT in isolated mitochondria by Mm C can be abolished by cyclosporin A (Cs A) treatment. Mouse liver mitochondria incubated with Ca^2+^were treated with Mm C, Cs A or combination of Mm C and Cs A. Then absorbance was recorded. **(C)** Mm C dose-dependently induced ROS accumulation in isolated mitochondria. Mouse liver mitochondria incubated with Ca^2+^ were treated with indicated concentrations of Mm C and then fluorescence was recorded over 15 min. **(D)** Mm C-induced ROS accumulation in isolated mitochondria can be abolished by Cs A treatment. Mouse liver mitochondria incubated with Ca^2+^ were treated with Mm C, Cs A or combination of Mm C and Cs A. Then fluorescence was recorded. **(E)** Ca^2+^ is essential for Mm C-induced ROS accumulation in isolated mitochondria. Mouse liver mitochondria incubated with or without Ca^2+^ were treated with Mm C. Then fluorescence was recorded. **(F)** Cs A reduced Mm C-induced ROS production in A549 cells. A549 cells were pretreated with the indicated concentrations of Cs A for 1 h and then Mm C was added for another 4 h. The ROS levels were measured by flow cytometry. ^*^P < 0.05. **(G)** Cs A prevented the cytotoxic effect of Mm C. A549 cells were treated with Mm C, Cs A or Mm C together with Cs A for 24 h and cell viability was measured using the MTT method. ^***^P < 0.001. **(H)** Mm C and sorafenib synergized to induce cell death in A549 cells. A549 cells were treated with Mm C, sorafenib or Mm C together with sorafenib for 24 h and cell viability was measured using the MTT method. Combination index (CI) was calculated with CompuSyn software.

In addition to uncoupler, mitochondrial respiration can also be stimulated through opening of mitochondrial permeability transition pore (PTP). Opening of mitochondrial PTP causes unlimited proton movement across the inner mitochondrial membrane which results in uncoupling of oxidative phosphorylation. Since Ca^2+^ is essential for mitochondrial PTP opening, we tested the opening of mitochondrial PTP at different Ca^2+^ concentrations and chose the concentration of 20 μM in the following experiments (Supplementary Figure 4). Next, we examined whether Mm C could modulate mitochondrial PTP using isolated mouse liver mitochondria in isotonic sucrose solution supplied with 20 μM Ca^2+^. Opening of mitochondrial PTP was monitored by the changes of absorbance at 540 nm, which reflected mitochondrial permeabilization to sucrose. As shown in Figure 7A, Mm C dose-dependently induced opening of mitochondrial PTP as revealed by decrease in light scattering due to mitochondrial swelling. Also, as shown in Figure 7B, the induction of mitochondrial swelling by Mm C was inhibited by cyclosporine A (Cs A), an inhibitor of the mitochondrial PTP, indicating that mitochondrial swelling induced by Mm C was mediated through opening of mitochondrial PTP.

Since ROS is closely relevant to PTP opening, we tested whether Mm C could modify mitochondrial production of ROS indicated by the fluorescence changes of DCFH_2_-DA. As shown in Figure 7C, addition of DCFH_2_-DA to respiring mitochondria in swelling buffer with 20 μM Ca^2+^ led to linear increase in fluorescence over time, reflecting ROS formation due to basal mitochondrial respiration. Addition of Mm C dose-dependently increased the fluorescence (Figure 7C), indicating that Mm C directly induced ROS production in mitochondria. Additionally, Cs A reversed the increase of fluorescence induced by Mm C (Figure 7D), demonstrating that Mm C induced ROS production through opening of mitochondrial PTP.

Increased oxidative stress is one of the key mediators of compound-induced mitochondrial permeability transition (MPT), therefore, we assessed the causal relationship between the two effects of Mm C: the induction of MPT and mitochondrial ROS production. Since mitochondrial PTP opening is dependent on Ca^2+^, we examined whether the effects of Mm C on mitochondrial ROS production were dependent on the presence of Ca^2+^. As shown in Figure 7E, Mm C greatly increased fluorescence in the presence of Ca^2+^, while fluorescence induction was significantly weakened when Ca^2+^was deprived. These data indicated that ROS production was mainly a consequence of MPT induction.

Also, we confirmed the causal relationship between MPT and ROS production in A549 cells. As shown in Figure 7F, pretreatment of A549 cells with Cs A dose-dependently abolished Mm C-induced elevation of ROS, indicating that ROS production was primarily a consequence of MPT induction. Also, Cs A dose-dependently abrogated Mm C-mediated cytotoxic effect of A549 cells (Figure 7G). These data indicated that Mm C induced ROS production and cytotoxicity of A549 cells through induction of MPT.

Since cancer cells frequently have multiple genetic alterations, a combination of different anticancer agents might be required to effectively cancer therapy. We combined Mm C with multi-kinase inhibitor sorafenib as well as anti-microtubule drug paclitaxel and found that Mm C and sorafenib synergized to induce cell death in A549 cells (Supplementary Figure 5). As shown in Figure 7H, CI (combination index) <1, indicating synergy, was detected in the combination of Mm C and sorafenib at most concentrations of each drug. Since ROS inducer tetrandrine was reported to have synergistic antitumor effect with sorafenib through ROS/Akt signaling [41], the ROS production effect of Mm C probably contributed to the synergistic effect of Mm C and sorafenib. The synergistic effect of Mm C and sorafenib opens new opportunity to enhance the effectiveness of sorafenib in cancer treatment.

## DISCUSSION

In marine microorganisms, the class actinobacteria is especially notable for containing organisms producing diverse natural products. In actinomycets, the genus *Streptomyces* produces 70% to 80% of currently characterized actinomycete natural products [42]. Mm C is a cytotoxic isoquinolinequinone isolated from a marine streptomycete. However, the exact mechanisms of action of Mm C and most marine-derived cytotoxic isoquinolinequinones are still unknown. In the present study, we synthesized Mm C and investigated its molecular targets and mode of action. We demonstrate that Mm C is a novel inducer of MPT, which increases ROS production and preferentially kills cancer cells.

PTP opening appears to be accompanied by a burst of ROS [43, 44], and it is well known that oxidant stress is one of the key factors that favor induction of MPT [22], so it seems difficult to distinguish the primary cause to secondary effect of Mm C-induced PTP opening and ROS production of mitochondria. However, we have several lines of evidence suggesting that ROS production is mainly a consequence of MPT induction. Firstly, Mm C-induced ROS production in both mitochondria and A549 cells could be inhibited by Cs A, an inhibitor of the mitochondrial PTP. Secondly, Ca^2+^, which is the single most important factor for PTP opening *in vitro*, was essential for Mm C-induced mitochondrial ROS production. Although Mm C slightly increased mitochondrial ROS when Ca^2+^ was deprived, it is probably that the isolated mitochondria contain residual endogenous Ca^2+^. Lastly, a ubiquinone-binding site has been proven to regulate the mitochondrial PTP and ubiquinone has similar structure to Mm C, which all belong to quinones [45, 46]. We propose that Mm C targets mitochondrial PTP to cause MPT and results in mitochondrial ROS production, which in turn facilitates the opening of PTP [22]. Thus, we speculate that PTP opening induced by Mm C rapidly propagates to the whole cell, leading to a massive oxidative stress which preferentially kills cancer cells.

**Figure 8:**
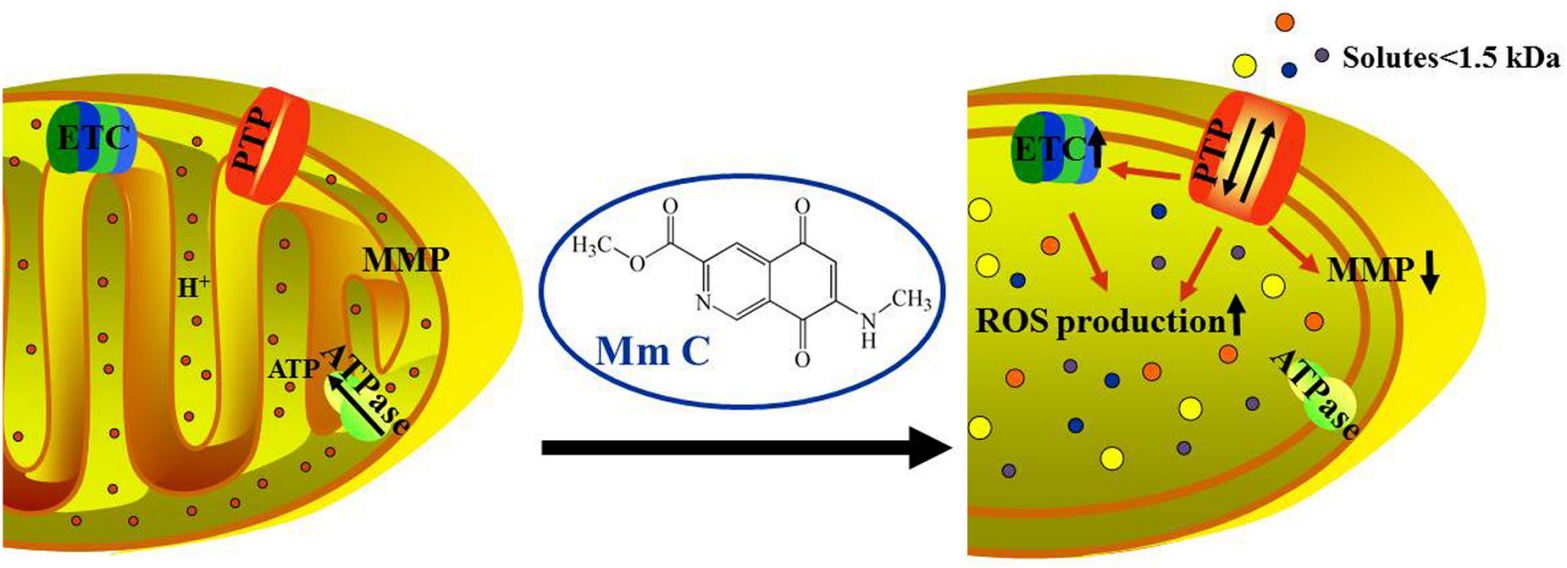
Proposed mechanism of Mm C After compound uptake, Mm C induces opening of mitochondrial PTP which causes ROS production, decrease of MMP and mitochondrial structural (enlarged mitochondria with cristal disorganization or loss) and functional (increased respiration without accompanying ATP production) defects. Mitochondrial functional defects stimulate expression of ETC proteins and cellular respiration, which in turn facilitate the production of ROS. The opening of mitochondrial PTP, accumulation of ROS and mitochondrial dysfunction finally lead to cell death. (In the figure, small red circles indicate hydrogen ions and circles with other colors indicate all molecules with molecular weight less than 1.5 kD.)

Mm C resulted in a 43-fold increase of ROS fluorescence intensity in A549 cells and the intensity of Mm C-induced ROS accumulation is much higher than that of other compounds [7–9]. Also, our proteomic results showed that many differentially expressed proteins were closely related to ROS, indicating ROS is the major effect in Mm C-treated cancer cells. Mm C stimulated the expression of ETC proteins and cellular respiration which may compensate for the decline of mitochondrial respiratory function, especially the decrease of intracellular ATP levels [47]. Mitochondrial ETC is the major sites of endogenous ROS production as a by-product of respiration [16], so the stimulated expression of ETC proteins and cellular respiration caused by Mm C further increased ROS production (Figure 8), accounting for the extremely high intensity of Mm C-induced ROS accumulation.

The opening of mitochondrial PTP induced by Mm C resulted in a sudden increase in permeability of the mitochondrial inner membrane, leading to dissipation of MMP and this in turn promoted the opening of PTP [48]. Therefore, Mm C-induced MPT caused rapid and expanding decrease of MMP, which stimulated uncoupled respiration and decreased intracellular ATP levels and finally caused mitochondrial dysfunction.

It is difficult to determine the exact order of effects of Mm C in cancer cells for that these effects entangle together and influence each other. However, we hypothesize that the primary target of Mm C is mitochondrial PTP. As shown in Figure 8, the opening of PTP caused by Mm C induces ROS production as well as mitochondrial dysfunction and these two events promote each other to preferentially kill cancer cells.

In summary, our data reveals that Mm C, a marine-derived natural compound isolated from *Streptomyces* sp. isolate Mei37, preferentially kills cancer cells through mitochondrial PTP opening-mediated ROS production. Furthermore, Mm C acts synergistically with anticancer drug sorafenib. These data strongly indicate that Mm C is a promising anticancer drug candidate.

## MATERIALS AND METHODS

### Reagents

Mm C was synthesized by Shanghai SIMR Biotech. Co., Ltd (Shanghai, China) with a purity of 95.65% (Supplementary Figure 6). MTT, sorafenib, reduced glutathione (GSH) detection kit, BCA kit for protein quantification, intracellular ATP detection kit and JC-1 MMP detection kit were from Beyotime Institute of Biotechnology (Shanghai, China). Annexin V-FITC/PI apoptosis detection kit was from Nanjing KeyGEN Biotech. Co., Ltd. (Nanjing, Jiangsu, China). GSH, rotenone and cyclosporine A (Cs A) were from Solarbio Science & Technology Co., Ltd (Beijing, China). NAC was from Aladdin Biotech (Beijing, China). Carbonyl cyanide m-chlorophenyl hydrazone (CCCP) was from abcam (Cambridge, MA, USA). Valinomycin was from Sangon Biotech (Shanghai, China).

### Cell lines and culture

All cell lines were obtained from the American Type Culture Collection. A549 cells were grown in RPMI 1640 medium (from Gibico, Grand Island, NY, USA) supplemented with 10% FBS (from Gibico, Grand Island, NY, USA), 100 μg/ml streptomycin and 100 units/ml penicillin (from Hyclone, Logan, UT, USA). WI-38, HEK-293T, LO2, HeLa and Bel-7402 cells were cultured in Dulbecco’s modified Eagle medium (DMEM, from Gibico) supplemented with 10% FBS, 100 μg/ml streptomycin and 100 units/ml penicillin. All cell lines were cultured at 37°C in a humidified atmosphere of 5% CO_2_ and 95% air.

### Cell viability measurement

Cell viability was measured by MTT assay as well as trypan blue exclusion staining. Briefly, about 5000-12000 logarithmically growing cells were placed into 96-well plates and cultured for 24 h. Cells were then treated with vehicle control (DMSO) or compounds for 6 h or 24 h.

For MTT assay, 30 μl MTT (5 mg/ml) solution was added to the culture medium. After incubated for 3 h at 37°C, 100 μl ‘Triplex Solution’ (10% SDS-5% isobutanol-12 mM HCl) was added to each well for 16 h, and then absorbance was measured at 570 nm by a spectrophotometer (Infinite M1000 Pro, TECAN, Mannedorf, Switzerland).

For trypan blue exclusion staining, the trypan blue stock solution (0.4%, from Sigma-Aldrich, Saint Louis, MO, USA) was mixed 1: 9 with cell suspension. Cells were then counted manually using a hemocytometer under microscope.

### ROS analysis

Mm C-induced ROS generation was determined by 2’, 7’-dichlorofluorescin diacetate (DCFH_2_-DA, from Sigma-Aldrich) as previous described [8]. Briefly, A549 cells were treated with 10 μM DCFH_2_-DA at 37 °C for 30 min before further analysis by flow cytometry (FACS Aria II, BD, San Jose, California, USA). For mitochondria (1 mg/ml), 10 μM DCFH_2_-DA were added to Ca^2+^ induced swelling buffer (as shown below in the Ca^2+^ induced mitochondrial swelling assay section) and fluorescence (488 nm excitation, 525 nm emission) was recorded over 15 min by a spectrophotometer [49]. Means and standard errors of 3 replicates per point are shown.

### Proteomic analysis

Proteomic analysis was performed by PTM Biolab, Inc. (Hangzhou, Zhejiang, China). Briefly, A549 cell were treated with 1 μM Mm C for 6 h or 12 h and proteins of whole-cell lysates were extracted, separated and digested. Peptides were then separated and identified by liquid chromatography (LC)-electron spray ionization (ESI)-tandem mass spectrometry (MS/MS) analysis.

The resulting MS/MS data were processed using Mascot search engine. Tandem mass spectra were searched against Swiss-Prot human database. For peptide search, trypsin/P was specified as cleavage enzyme allowing up to 2 missing cleavages. Mass accuracy was set to 10 ppm for precursor ions and 0.02 Da for fragment ions. Carbamidomethylation on Cys, TMT-6Plex on peptides N-term and Lys were included as fixed modification and oxidation on Met was specified as a variable modification. For result export, peptide ion score was set ≥20. For protein quantitation, a protein was required to contain at least two unique peptides. Protein quantitative ratios were weighted and normalized relative to the median ratio in Mascot. Only proteins with significant quantitative ratios between the two treatments (P< 0.05) and with fold changes ≥1.2 or ≤0.83 were considered to be differentially expressed.

For KEGG (Kyoto Encyclopedia of Genes and Genomes) pathway-based enrichment analysis of differentially expressed proteins, KEGG database was used to identify enriched pathways by a two-tailed Fisher’s exact test to test the enrichment of the differentially expressed proteins against all identified proteins. Correction for multiple hypothesis testing was carried out using standard false discovery rate control methods.

### Isolation of mouse liver mitochondria

Female ICR mice weighing approximately 25 g were used in this study. All mice experiments in this study were approved by Institute of Oceanology Laboratory Animal Care and Ethics Committee in accordance with the animal care and use guidelines.

Mitochondria from ICR mouse liver were prepared according to standard differential centrifugation procedures at 1000 g, 10000 g and 7000 g at 4 °C for 10 min as described [49]. Isolation medium contained 250 mM sucrose, 20 mM HEPES, 10 mM KCl, 1.5 mM MgCl_2_ and 1 mM EDTA (wash medium without EDTA) at pH 7.4.

### Respiration of cells and mitochondria

Oxygen consumption rate was polarographically monitored by an oxygraph (Hansatech, Norfolk, UK). The respiration medium of A549 cells (1.5×10^6^ cells/ml) was RPMI 1640 medium. While mitochondria (1.5 mg/ml) were incubated in respiration medium containing 125 mM sucrose, 10 mM HEPES, 65 mM KCl, 10 mM KH_2_PO_4_, 0.1 mM EDTA, 5 mM glutamate and 5 mM malate at pH 7.4 [49].

### Mitochondrial swelling assay in isotonic potassium acetate

To test the protonophoric properties of compounds, mitochondrial swelling assay was measured in isotonic potassium acetate as previously described [50]. Briefly, Mm C or CCCP were added into 100 μl isotonic potassium acetate medium (145 mM potassium acetate, 5 mM Tris, 0.5 mM EDTA, 1 μM rotenone and 1 μM valinomycin at pH 7.4) in 96-well transparent plate. Then mitochondria (1 mg/ml) were resuspended with 100 μl isotonic potassium acetate medium and added in 96-well transparent plate. Absorbance of mitochondrial suspension at 600 nm was recorded immediately with shaking over 10 min by a spectrophotometer. Means and standard errors of 3 replicates per point are shown.

### Ca^2+^ induced mitochondrial swelling assay

PTP opening was monitored by Ca^2+^ induced mitochondrial swelling assay as previous described [51]. Briefly, Mm C or Cs A or Mm C together with Cs A were added into a medium (250 mM sucrose, 10 mM HEPES, 6 mM succinate, 2.5 μM rotenone and 40 μM CaCl_2_ at pH 7.4) in 96-well transparent plate. Then mitochondria (1 mg/ml) were resuspended with 100 μl medium (250 mM sucrose, 10 mM HEPES, 6 mM succinate and 2.5 μM rotenone at pH 7.4) and added in 96-well transparent plate. Absorbance of mitochondrial suspension at 540 nm was recorded immediately with shaking over 10 min by a spectrophotometer. Means and standard errors of 3 replicates per point are shown.

### Statistical analysis

All data were shown only if three independent experiments showed consistent results. Data were graphically represented as mean ± SEM. Treatment means were compared by one-way analysis of variance followed by Tukey's test using GraphPad Prism software (San Diego, CA, USA). P values < 0.05 were considered statistically significant (^*^ p < 0.05, ^**^ p < 0.01, ^***^ p < 0.001).

## SUPPLEMENTARY MATERIALS FIGURES AND TABLES




