# Supplementary material for: Mansouramycin C kills cancer cells through reactive oxygen species production mediated by opening of mitochondrial permeability transition pore
Source: Oncotarget. 2017 Oct 24;8(61):104057–71. doi: 10.18632/oncotarget.22004 (PMC5732787; doi:10.18632/oncotarget.22004)
Supplement: Supplementary file 1 [file oncotarget-08-104057-s001.pdf]

Mansouramycin C kills cancer cells through reactive oxygen species production mediated by opening of mitochondrial permeability transition pore

SUPPLEMENTARY MATERIALS

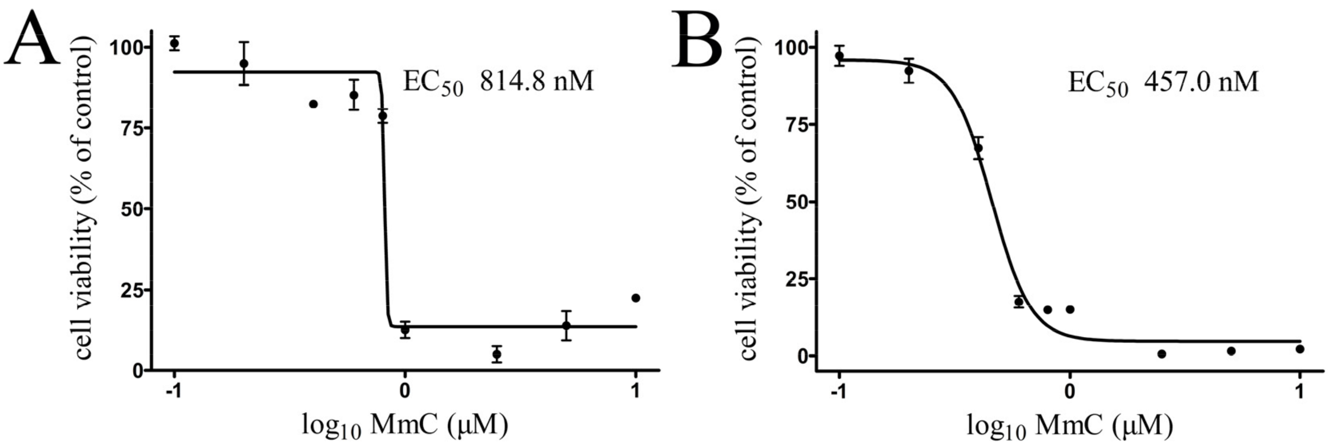

Supplementary Figure 1: EC_{50} of Mm C on viability of A549 cells. **(A)** EC_{50} of Mm C on viability of A549 cells for 6 h determined by trypan blue exclusion staining assay. **(B)** EC_{50} of Mm C on viability of A549 cells for 6 h and wash out of Mm C for another 24 h. A549 cells were treated with Mm C for 6 h, washed with fresh medium for three times and then incubated with fresh medium for another 24 h. EC_{50} of Mm C on cell viability was determined by MTT assay.

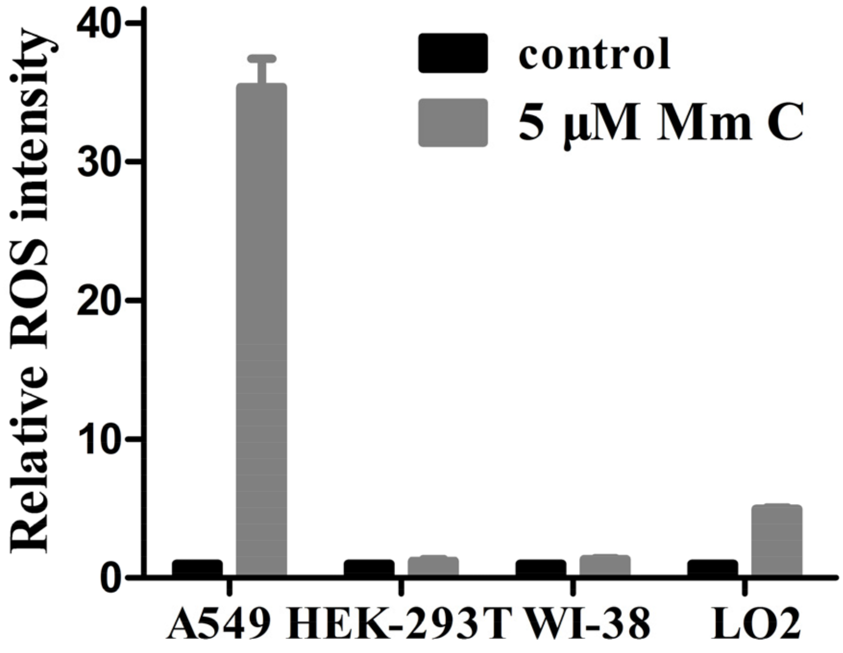

Supplementary Figure 2: Distinct ROS induction effects of Mm C on cancer cell line A549 and normal cell lines HEK-293T, WI-38 and LO2. A549, HEK-293T, WI-38 and LO2 cells were treated with 5 μM Mm C for 6 h and then ROS levels were measured by flow cytometry.

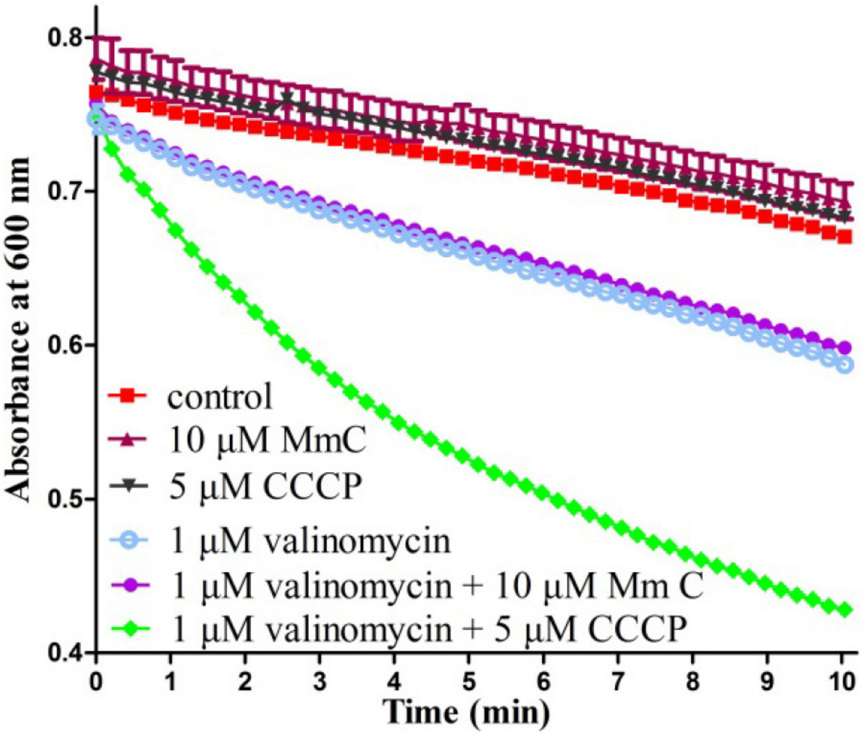

Supplementary Figure 3: Valinomycin is essential for proton-dependent mitochondrial swelling induced by carbonyl cyanide m-chlorophenyl hydrazone (CCCP). Isolated mouse liver mitochondria were treated with Mm C or CCCP in hyposmotic potassium acetate medium containing 1 μM valinomycin or not and then absorbance at 600 nm was recorded over 10 min.

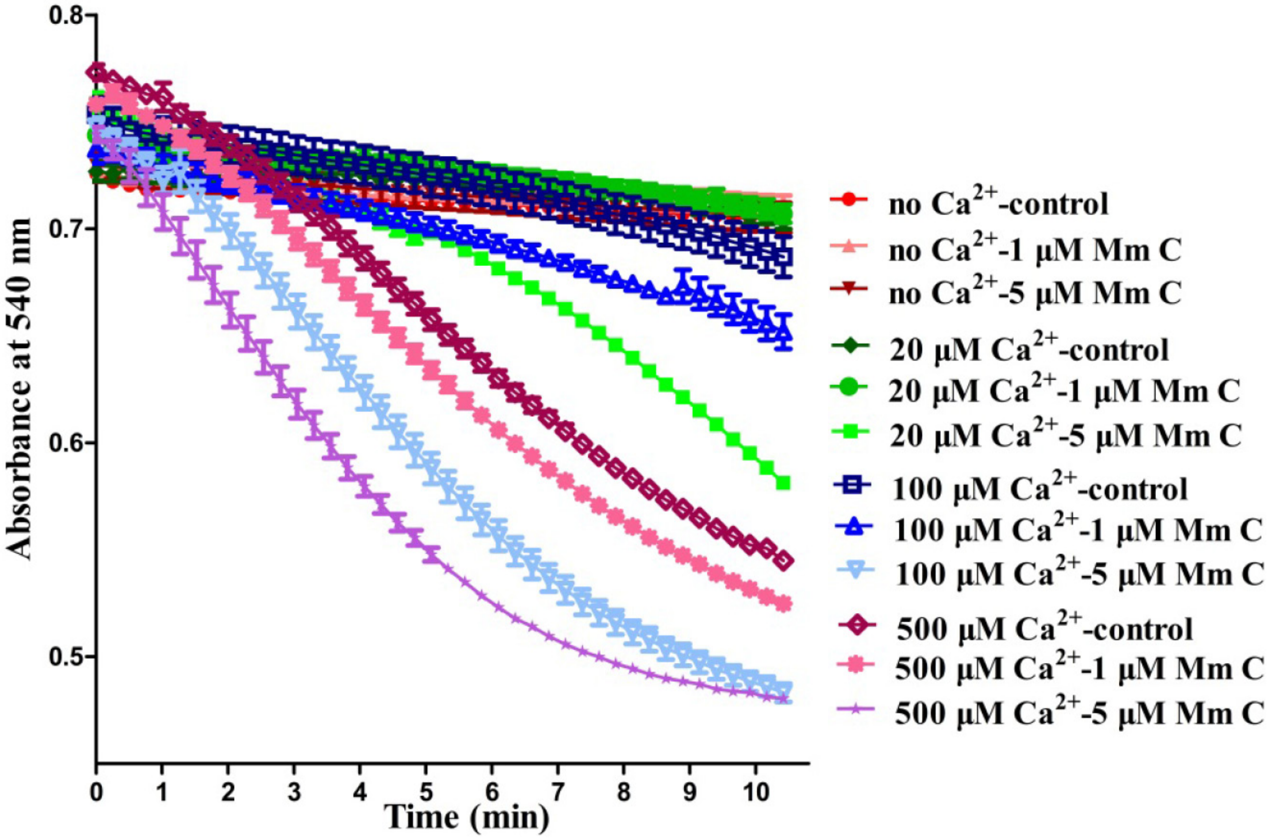

Supplementary Figure 4: Mm C dose-dependently induced mitochondrial permeability transition (MPT) under different concentrations of Ca^{2+}. Mouse liver mitochondria incubated with 0, 20, 100 and 500 μM Ca^{2+} were treated with indicated concentrations of Mm C and then absorbance at 540 nm was recorded over 10 min.

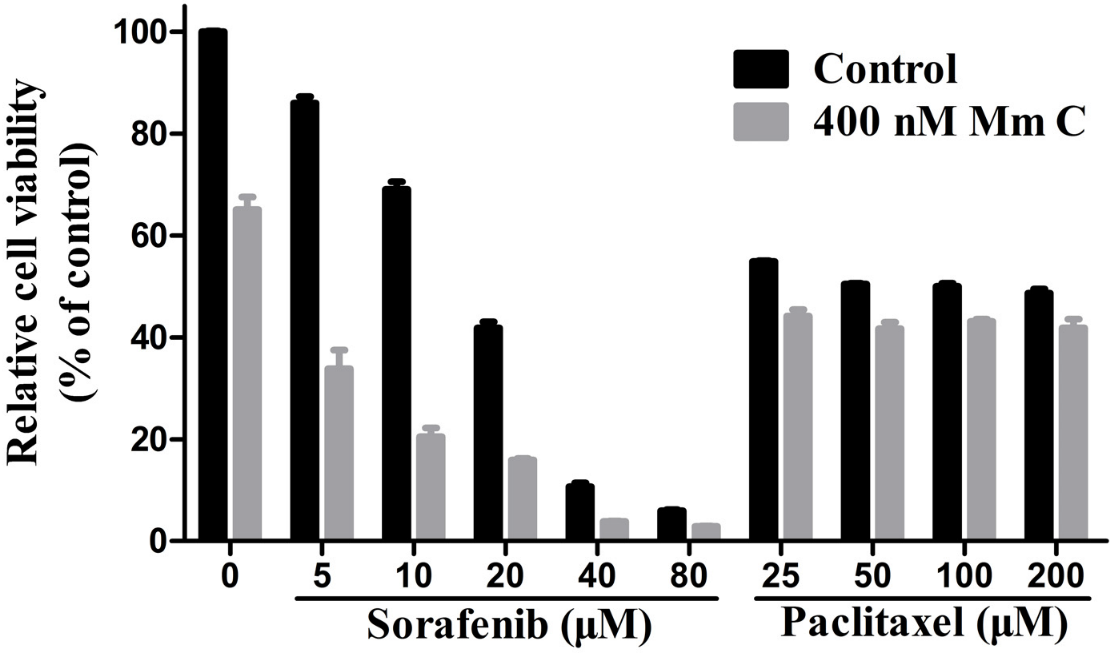

Supplementary Figure 5: Cytotoxic effects of Mm C combined with sorafenib or paclitaxel. A549 cells were treated with Mm C, sorafenib, paclitaxel, Mm C together with sorafenib or Mm C together with paclitaxel for 48 h and cell viability was measured using the MTT method.

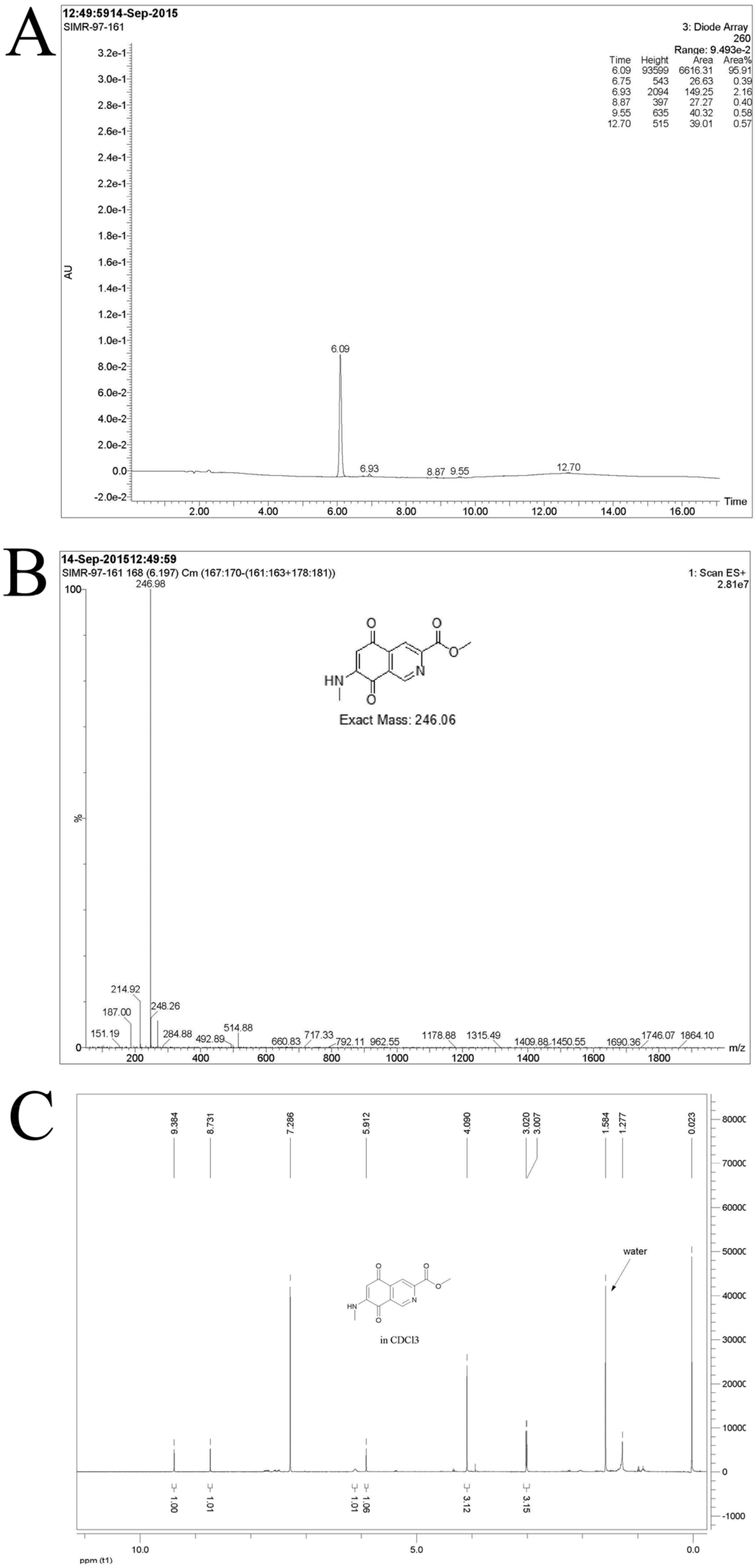

Supplementary Figure 6: HPLC, ESI-MS and 1H NMR spectrum of Mm C. **(A)** HPLC spectrum of Mm C. **(B)** ESI-MS spectrum of Mm C. **(C)** 1H NMR spectrum of Mm C.

Supplementary Table 1: Differentially expressed proteins after 1 µM Mm C treatment for 6 h

See Supplementary File 1

Supplementary Table 2: Differentially expressed proteins after 1 µM Mm C treatment for 12 h

See Supplementary File 1
